# Herbicidal efficacy of harzianums produced by the biofertilizer fungus, *Trichoderma brevicompactum*

**DOI:** 10.1186/s13568-020-01055-x

**Published:** 2020-07-01

**Authors:** Miaomiao Yin, Opemipo Esther Fasoyin, Chen Wang, Qun Yue, Yunyun Zhang, Baoqing Dun, Yuquan Xu, Liwen Zhang

**Affiliations:** 1 Biotechnology Research Institute, The Chinese Academy of Agricultural Sciences, 12 Zhongguancun South Street, Beijing, 100081 People’s Republic of China; 2 Institute of Crop Sciences, The Chinese Academy of Agricultural Sciences, 12 Zhongguancun South Street, Beijing, 100081 People’s Republic of China

**Keywords:** *Trichoderma brevicompactum*, Harzianum, *Brassica chinensis*, *Echinochloa crusgalli* L. Beauv., Phytotoxicity

## Abstract

Herbicides are important tools for weed control in modern agriculture. In the search for potential herbicidal natural products from fungal species, harzianum A and B were identified from the biofertilizer fungus, *Trichoderma brevicompactum*. In the phytotoxicity assays on the dicot species *Brassica chinensis*, harzianum A and B reduced both shoot and root lengths at low concentrations and inhibited the seed germination at 2 μg mL^−1^. In addition, harzianum A and B also exhibited phytotoxicity against monocots, *Oryza sativa* L. cv. Nipponbare and *Echinochloa crusgalli* L. Beauv.. Compared with a common herbicide, 2,4-dichlorophenoxyacetic acid, harzianum A and B performed similar activity in a pot assay, and were more effective in post-emergence than pre-emergence conditions. Harzianum A and B have potential as efficient herbicide for controlling important dicotyledon and monocotyledon weeds at low concentrations. They can be sprayed in liquid form in both pre- and post-emergence conditions. Our results confirmed the importance of these molecules for the development of new herbicides.

## Introduction

Weeds cause significant negative impact on crop productivity and consequent economic losses due to their competition with crops for soil, water and nutrients (Charudattan 2001; Rajcan and Swanton 2001; Tilman et al. 2002). Cultural, mechanical, chemical and biological methods are the most prominent approaches used for weed management. Among these, chemical herbicides remain the most effective method to date, because of their ease of application and greater accessibility for farmers; the dependence on these chemicals is increasing in recent decades, especially in emerging countries (Araniti et al. 2019). However, the intensive and indiscriminate application of these chemicals, especially those that are very persistent in agricultural end-products and not easily biodegradable, is one of the major causes of environmental pollution and human health threat. In addition, the rapid evolution of weed resistance calls for urgent need to develop new herbicides with high biological activity, as well as low or no toxicity, compared to the commercial herbicides (Araniti et al. 2019; Dayan and Duke 2014). Microbial natural products have long been playing important roles in medicine and agriculture; more than half of all small molecule drugs approved for use are derived from natural products, a large portion of which comes from microorganisms (Newman and Cragg 2020). However, until now, natural products have much less contribution in herbicide development compared to other pesticides (Copping and Duke 2007; Huter 2011), although many natural products and derivatives have been reported with herbicidal activity (Araniti et al. 2015, 2019; Duke et al. 2002; Mitchell et al. 2001; Zhou et al. 2019).

Fungi of the genus *Trichoderma* are commonly found in nature, including soil, water, household dust, foods, and decaying organic material, and are widely used as biological control agents for fungal phytopathogens and for the production of enzymes (Harman et al. 2004). *Trichoderma* spp. produce a number of natural products belonging to several classes of chemicals with different biological activities, such as antibiotic peptides and peptaibols, volatile pyrones and lactones, plant growth factors, siderophores, etc. (Benitez et al. 2004; Vinale et al. 2008). They also produce the mycotoxins, trichothecenes (Corley et al. 1994; Nielsen et al. 2005). Trichothecenes are a group of sesquiterpenoid-derived natural products with various patterns of oxygenations and esterifications of a core tricyclic structure with an epoxide function (Cardoza et al. 2011).

Trichothecenes are generally phytotoxic. They can cause necrosis, chlorosis and mortality to plants, enabling them to mediate a wide variety of plant diseases, including wilts, stalk rot, root rot and leaf rot in many important crop and ornamental plants, by binding to 60s ribosomes and interrupting protein synthesis in eukaryotic cells, which makes them potential herbicides (Bin-Umer et al. 2011; McCormick et al. 2011; Nishiuchi et al. 2006; Tijerino et al. 2011; Wang et al. 2006). However, this potential was hindered by their toxicity to humans and livestock e.g., vomiting, alimentary hemorrhaging, and dermatitis (McCormick et al. 2011). Nevertheless, this toxicity is highly dependent on the structure. Trichothecenes can be divided into four types according to functional groups. Type A has a functional group other than a keto group at C-8. This is the largest group and includes toxins like T-2 toxin. Type B trichothecenes have a keto group at C-8 and include the most widespread trichothecene deoxynivalenol. Type C has a second epoxide ring at C-7,8 or C-9,10 and toxins from Type D contain a macrocyclic ring between C-4 and C-15 with two ester-linkages. Simple trichothecenes including type A and type B are generally less toxic than macrocyclic trichothecenes (Abbas et al. 2013). In addition, peracetylation of type B trichothecenes and de-epoxidation of type A trichothecenes both substantially reduced mammalian toxicity with little effect on phytotoxicity (Abbas et al. 2013). Thus, type A and type B trichothecenes and their derivatives can be potential bioherbicide candidates as long as they possess high phytotoxicity and low mammalian toxicity. One of the type A trichothecenes, harzianum A (HA) consists of the core trichothecene structure (12,13-epoxytrichothec-9-ene, EPT) with a linear polyketide-derived substituent (octa-2,4,6-trienedioyl) esterified to an oxygen at carbon atom, and was first isolated from *Trichoderma harzianum* and then *T. brevicompactum* (Cardoza et al. 2019; Corley et al. 1994; Nielsen et al. 2005). Its *cis*–*trans* isomer, harzianum B (HB) was later identified from *Hypocrea* (teleomorph of *Trichoderma*) sp. F000527 (Jin et al. 2007). Both compounds were tested for cytotoxicity against HeLa, MCF-7, and HT1080 cell lines (Jin et al. 2007), but their phytoxicity was unknown.

In our search for potential herbicidal leading compounds from fungal strains, we identified HA and HB (**1**–**2**) from the biofertilizer fungus, *T. brevicompactum* (CGMCC19618). The herbicidal efficacy of HA and HB (**1**–**2**) was assayed on the dicot *Brassica chinensis*, monocot *Oryza sativa* L. cv. Nipponbare, and monocot weeds (*Setaria viridis* L. Beauv. and *Echinochloa crusgalli* L. Beauv.). The common commercial herbicide, 2,4-dichlorophenoxyacetic acid (2,4-D) was used as positive control. The results revealed the potential of HA and HB as herbicide.

## Materials and methods

### Screening of herbicidal compounds using *B. chinensis*

The dicot *B. chinensis* was selected for phytotoxicity testing to screen the fungal strains that produce herbicidal compounds. Briefly, 5 high-quality seeds were transferred to each well of the 96-well plate, with 50 μL water containing crude extract or purified compounds in the concentration range 0.2–2 mg mL^−1^ or 5–25 nM. Cultures were incubated at 25 °C under light/dark cycle of 16 h/8 h in a growth chamber. Each condition was triplicated and repeated at least three times. The germination rate was examined for visual signs of phytotoxicity. 2,4-Dichlorophenoxyacetic acid (2,4-D) was used as positive control.

### Isolation and identification of harzianum A and B

The fungal strain used in this study was *T. brevicompactum* CGMCC19618. Preparation of fermentation extracts, analysis by liquid chromatography-mass spectrometry (LC–MS). HPLC-HRESIMS and MS–MS spectra were acquired on an Agilent 1290 Infinity II HPLC coupled with an Agilent QTOF 6530 instrument. ^1^H and ^13^C NMR spectra were obtained on an Agilent DD2 spectrometer at 600 MHz for ^1^H NMR and 150 MHz for ^13^C NMR. Two-liter fermentation broth was extracted using ethyl acetate. The extracts were first subjected to silica gel (25 g) column chromatography and eluted with a gradient of chloroform/methanol to yield five fractions (Fraction A, v/v 100:0, 250 mL; Fraction B, v/v 99:1, 250 mL; Fraction C, v/v 98:2, 250 mL; Fraction D, v/v 95:5, 250 mL; Fraction E, v/v 90:10, 250 mL). Each of these fractions was analyzed by HPLC–MS. Fractions containing the target compounds were subsequently purified by semi-preparative HPLC on an Agilent Eclipse XDB-C18 reversed-phase column (5 mm, 9.4 mm × 250 mm) using an Agilent 1260 Infinity II system.

Harzianum A (**1**): ^1^H NMR (400 MHz, methanol-*d*_4_) *δ* 7.90 (1H, dd, *J* = 15.1, 11.6 Hz, H-4′), 7.39 (1H, dd, *J* = 15.3, 11.3 Hz, H-6′), 6.78 (1H, t, *J* = 11.4 Hz, H-3′), 6.71 (1H, dd, *J* = 15.0, 11.3 Hz, H-5′), 6.07 (1H, d, *J* = 15.3 Hz, H-7′), 5.82 (1H, d, *J* = 11.4 Hz, H-2′), 5.70 (1H, dd, *J* = 7.9, 3.5 Hz, H-4), 5.38 (1H, d, *J* = 5.3 Hz, H-10), 3.78 (1H, d, *J* = 5.2 Hz, H-2), 3.70 (1H, d, *J* = 5.3 Hz, H-11), 3.11 (1H, d, *J* = 3.9 Hz, H-13α), 2.91 (1H, d, *J* = 3.9 Hz, H-11β), 2.57 (1H, dd, *J* = 15.4, 7.8 Hz, H-3α), 2.04 (1H, m, H-3β), 2.00 (2H, m, H-8), 1.94 (1H, m, H-7β), 1.71 (3H, s, H-16), 1.48 (1H, m, H-7α), 0.97 (3H, s, H-15), 0.73 (3H, s, H-14); ^13^C NMR (100 MHz, methanol-*d*_4_) *δ* 169.8 (C-8′), 167.1 (C-1′), 145.0 (C-6′), 144.3 (C-3′), 141.2 (C-9), 139.4 (C-5′), 136.9 (C-4′), 126.1 (C-7′), 121.5 (C-2′), 119.8 (C-10), 80.4 (C-2), 76.6 (C-4), 72.0 (C-11), 66.6 (C-12), 50.5 (C-5), 48.7 (C-13), 41.5 (C-6), 37.6 (C-3), 28.9 (C-8), 25.5 (C-7), 23.3 (C-16), 16.3 (C-15), 6.5 (C-14); (+)-HRESIMS *m/z* 401.2003 [M+H]^+^ (calcd. for C_23_H_29_O_6_, 401.1964).

Harzianum B (**2**): ^1^H NMR (400 MHz, methanol-*d*_4_) *δ* 7.40 (1H, dd, *J* = 15.4, 10.0 Hz, H-6′), 7.34 (1H, dd, *J* = 15.4, 10.0 Hz, H-3′), 6.79 (2H, d, *J* = 10.0 Hz, H-4′ and H-5′), 6.11 (1H, d, *J* = 15.4 Hz, H-7′), 6.07 (1H, d, *J* = 15.4 Hz, H-2′), 5.69 (1H, dd, *J* = 7.9, 3.5 Hz, H-4), 5.38 (1H, d, *J* = 5.3 Hz, H-10), 3.75 (1H, d, *J* = 5.2 Hz, H-2), 3.70 (1H, d, *J* = 5.3 Hz, H-11), 3.10 (1H, d, *J* = 3.9 Hz, H-13α), 2.90 (1H, d, *J* = 3.9 Hz, H-11β), 2.55 (1H, dd, *J* = 15.4, 7.8 Hz, H-3α), 2.04 (1H, m, H-3β), 2.00 (2H, m, H-8), 1.94 (1H, m, H-7β), 1.71 (3H, s, H-16), 1.48 (1H, m, H-7α), 0.96 (3H, s, H-15), 0.72 (3H, s, H-14); ^13^C NMR (100 MHz, methanol-*d*_4_) *δ* 169.8 (C-8′), 167.7 (C-1′), 144.9 (C-6′), 144.5 (C-3′), 141.2 (C-9), 138.8 (C-5′), 138.2 (C-4′), 126.2 (C-7′), 125.2 (C-2′), 119.8 (C-10), 80.5 (C-2), 76.8 (C-4), 72.0 (C-11), 66.6 (C-12), 50.5 (C-5), 48.6 (C-13), 41.6 (C-6), 37.4 (C-3), 28.9 (C-8), 25.5 (C-7), 23.3 (C-16), 16.3 (C-15), 6.3 (C-14); (+)-HRESIMS *m/z* 401.2003 [M+H]^+^ (calcd. for C_23_H_29_O_6_, 401.1964).

### Phytotoxicity assays of harzianum A and B

The seeds of *B. chinensis*, *O. sativa* L. cv. Nipponbare, *S. viridis* L. Beauv. or *E. crusgalli* L. Beauv. were surface sterilized in 0.5% NaOCl (3 min), and then rinsed six times in distilled water. 1 μL of HA and HB (**1**–**2**) was applied to five seeds at doses from 1 ng to 100 μg per seed (Vinale et al. 2009). Equal volume of methanol was added to the control seeds. The treated seeds were sown in Petri dishes (100 × 150 mm) containing agar medium (0.8% w/v). Plates were placed vertically in the growth chamber (25 ± 2 °C temperature, 55% HR, and 16 h/8 h light/dark circle) to promote geotropic root growth. After treatment, seedlings were collected and separated into shoot and root. Growth inhibition was measured as reduced root or shoot length relative to the negative control (1 μL methanol). 2,4-D was used as positive control in all experiments. Each treatment was repeated two times.

### Pot assays of harzianum A and B

The herbicidal activity of HA and HB (**1**–**2**) mixture was tested against the dicot *B. chinensis* in pot assays with 2,4-D as positive control. The test compounds were dissolved in 10 μL methanol and diluted with 5 mL water to the required concentrations, and applied to pot-grown plants in a greenhouse. Plastic pots with a diameter of 9.5 cm were filled with garden soil to a depth of 8 cm. Approximately 15 seeds of the tested weeds were sown in the soil at a depth of 1–2 cm and grown at a temperature of 25 °C in a greenhouse. The air relative humidity was 50%. For pre-emergence assays, 5 mL of the HA and HB solution was sprayed to the soil. For post-emergence assays, the *B. chinensis* was treated at the two-leaf stage with designated dosages with three replicates. The solvent (10 μL methanol in 5 mL water) was used as negative control. Herbicidal activity was evaluated visually 6 days post treatment.

## Results

### Identification of the herbicidal compounds, harzianum A and B

In the search for herbicidal compounds, crude extracts of fungal strains isolated from soil were tested using *B. chinensis*. One crude extract completely inhibited germination at a concentration of 0.2 mg mL^−1^. This extract was from a fungal strain that belongs to the species *T. brevicompactum*.

The herbicidal compounds were identified from the crude extract by bioactivity-oriented isolation. Filtered culture broth of *T. harzianum* was extracted exhaustively with ethyl acetate. The reddish-brown residue recovered was subjected to silica gel with gradient elution. The phytotoxic fraction was purified by semi-preparative HPLC to yield a mixture of two known compounds HA (**1**) and HB (**2**), which are geometrical isomers with an estimated ratio of 3:1 according to the integration of ^1^H NMR spectrum. **1** and **2** belong to type A trichothecene and consist of the core trichothecene structure (12,13-epoxytrichothec-9-ene, EPT) connected with a linear polyketide-derived substituent (octa-2,4,6-trienedioyl) via an ester bond at C-4. Their HRMS and NMR spectroscopic data are consistent with reported values (see “Experimental” section) (Jin et al. 2007).
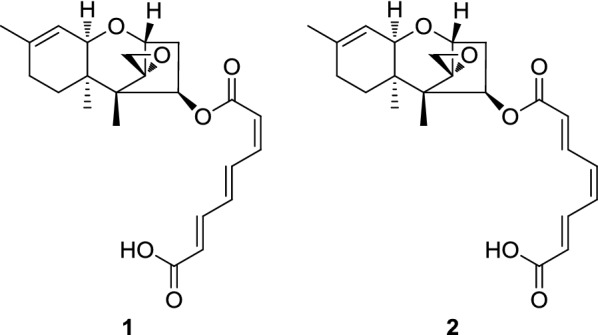


### Phytotoxicity of harzianum A and B to *B. chinensis*

**Fig. 1:**
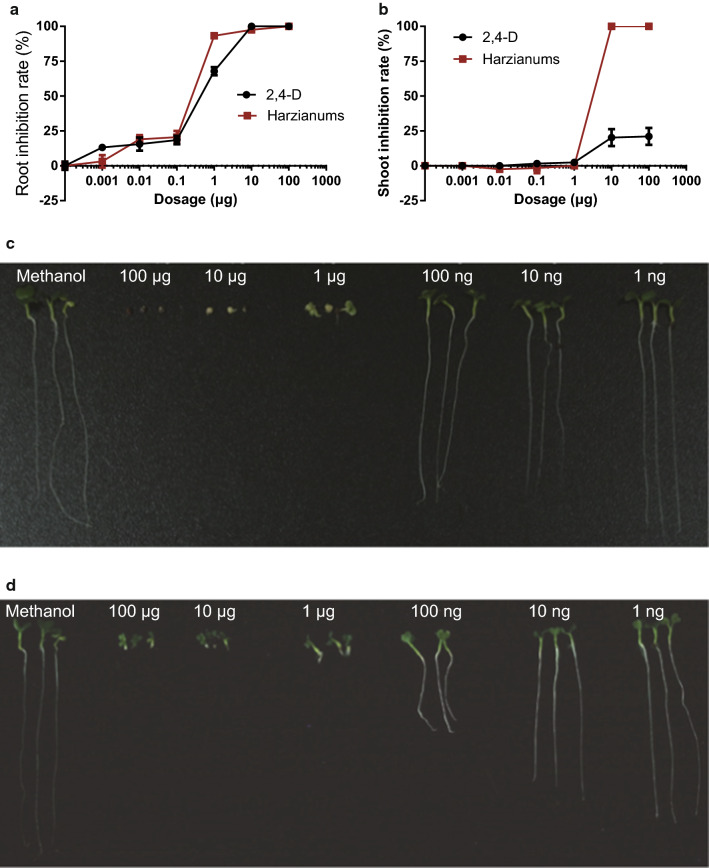
The phytotoxic effect of HA and HB (**1**–**2**) on *B. chinensis* compared to the positive control, 2,4-D. The inhibition rate of **a** root or **b** shoot length by HA and HB or 2,4-D (n = 5). The photographs of *B. chinensis* seedlings after treatment with **c** HA and HB or **d** 2,4-D

HA and HB (**1**–**2**) caused a strong inhibitory effect on the germination of *B. chinensis* seeds at a concentration of 5 μM. In general, the performances of HA and HB were comparable to the positive control, 2,4-D. When directly applied to the seeds at low concentrations (1–100 ng), the compounds caused significant root length reduction with inhibition values ranging from 3.2% (1 ng) to 20.6% (100 ng) at p values < 0.05 (Fig. 1a). However, the shoot length was not affected at these dosages. As the concentration increased (≥ 1 μg), the root growth inhibition by HA and HB (**1**–**2**) dramatically increased to 93.3%, which was higher than the value of 2,4-D (67.9%). When the concentration was increased to 10 μg, failure of development was observed on both shoot and root (Fig. 1a, b). In addition, the cotyledons appeared to be depigmented (Fig. 1c). In comparison, the positive control, 2,4-D, induced a significant shoot growth reduction at concentrations higher than 10 μg, with inhibition values ranging from 20.3 and 20.1%, respectively, and depigmentation was not observed (Fig. 1).

**Fig. 2:**
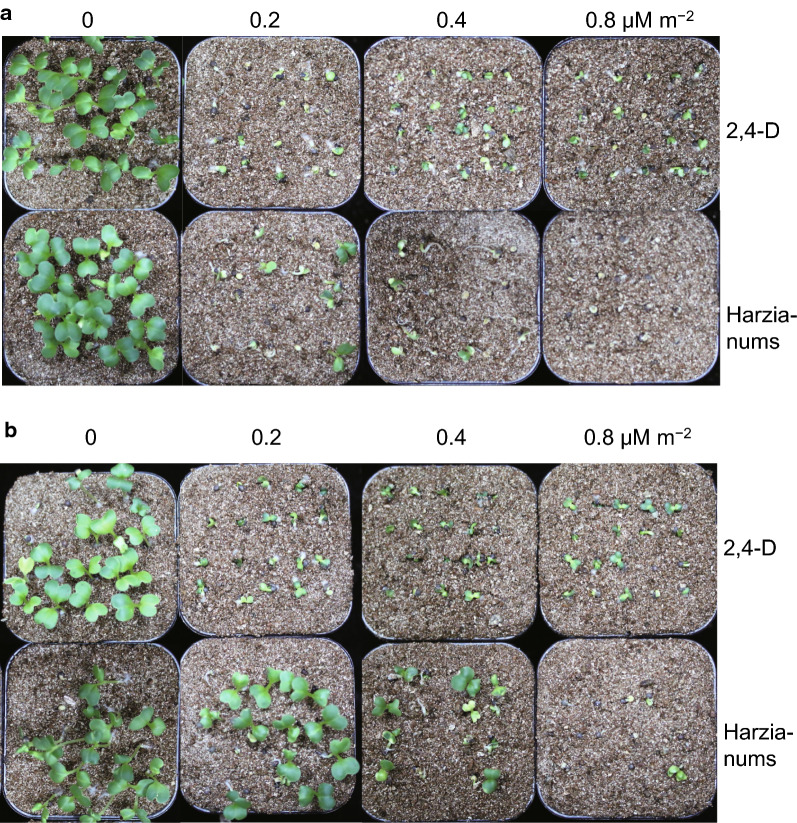
The herbicidal activities of HA and HB (**1**–**2**) on *B. chinensis* in pot assays in **a** pre- and **b** post-emergence conditions

The herbicidal activities of HA and HB (**1**–**2**) to *B. chinensis* were further evaluated in a greenhouse with 2,4-D used as positive control. The compounds were tested at dosages of 0.2, 0.4, and 0.8 μM m^−2^ for pre-emergence and post-emergence herbicidal activity. When sprayed after germination, harzianums showed good herbicidal activity at all three dosages at 6 days, which was comparable to 2,4-D (Fig. 2a). The seedling survival rate of *B. chinensis* was 35–60% for the harzianums, much lower than that of 2,4-D. In contrast, when applied to the soil before germination, the performance of harzianums was not as good as 2,4-D (Fig. 2b), and the germination was not significantly affected.

### Phytotoxicity of harzianum A and B to monocots

**Fig. 3:**
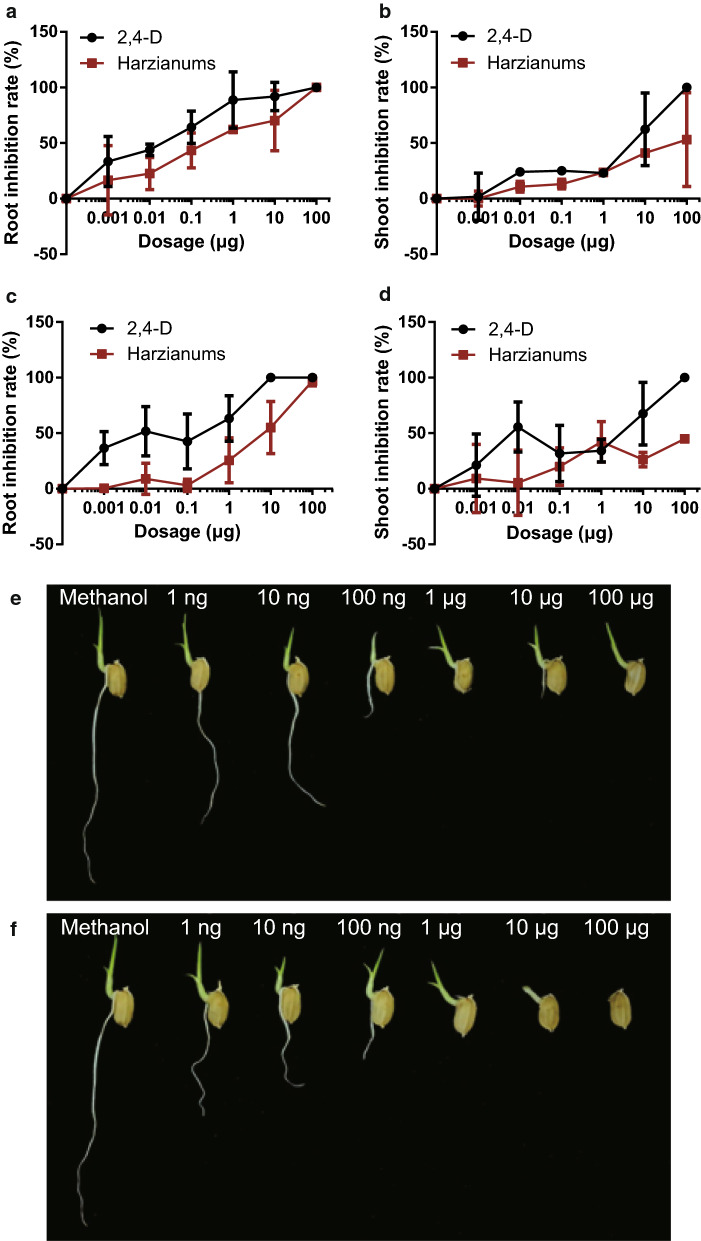
The phytotoxic effect of HA and HB (**1**–**2**) on rice or *E. crusgalli* L. Beauv. compared to the positive control, 2,4-D. The inhibition rate of **a**, **c** root or **b**, **d** shoot length by HA and HB or 2,4-D to **a**, **b** rice or **c**, **d***E. crusgalli* L. Beauv., respectively (n = 5), and the photographs of rice seedlings after treatment with **e** HA and HB or **f** 2,4-D

To determine the crop/weed selectivity, the phytotoxicity assays of HA and HB (**1**–**2**) were carried out on the monocots, i.e., rice (*O. sativa* L. cv. Nipponbare) and two weeds (*S. viridis* L. Beauv. and *E. crusgalli* L. Beauv.). The herbicidal effectiveness was compared with that of 2,4-D based on germination and biomass production (shoot and root length), at dosage ranging from 1 ng to 100 μg. The most significant reduction by HA and HB was observed on rice (Fig. 3). Significant root length reduction (16.51–100%, p < 0.05) was observed at the concentration range from 1 ng to 100 μg, though it was lower, compared to that caused by commercial herbicide (33.4–100%). This inhibitory effect was also evident, at lower extent, on *E. crusgalli* L. Beauv., resulting in a reduction of 25.5, 55.1, and 96.9% at concentrations of 1, 10 and 100 μg, respectively. In general, the phytotoxicity of HA and HB was less effective than that of 2,4-D to the root development of rice and *E. crusgalli* L. Beauv. (Fig. 3a, c), with IC_50_ values of 42.9 ng and 63.0 ng for 2,4-D, and 186.2 ng and 9.16 μg for HA and HB. The shoot length was less affected by HA and HB with IC_50_ values larger than 100 μg, while 2,4-D confirmed greater phytotoxicity to shoot length development at concentrations of 10 and 100 μg for both rice and *E. crusgalli* L. Beauv. However, *S. viridis* L. Beauv. did not appear to be significantly affected by HA and HB or 2,4-D up to 10 μg dosage.

## Discussion

In the search for a potential natural product-based herbicide, harzianum A and B (HA and HB) were found to be produced by the biofertilizer fungus, *T. brevicompactum*. HA and HB can exert significant phytotoxic effects on both dicot and monocot, but varied in different species. The growth inhibition by HA and HB mainly occurred in the root system. When grown in soil, application of the compounds on soil before the seedling emergence was less effective than after. In summary, HA and HB exhibited potential to be herbicides.

HA was first isolated from *T. harzianum* and then *T. brevicompactum* (Cardoza et al. 2019; Corley et al. 1994; Nielsen et al. 2005), while HB was later identified from *Hypocrea* sp. F000527 (Jin et al. 2007). HB showed weak cytotoxicity against HeLa, MCF-7, and HT1080 cell lines with IC_50_ values of 74.18, 74.04 and 15.63 μg mL^−1^, while HA was more toxic with IC_50_ values of 5.07, 10.13 and 0.65 μg mL^−1^, respectively (Jin et al. 2007). It seemed that the *cis*–*trans*–*trans* stereochemistry of HA contributes to this cytotoxicity. Therefore, to further evaluate the herbicidal potential of HA and HB, it is necessary to test them individually.

## Data Availability

The datasets on which the conclusions of the manuscript rely to were presented in the main paper.
